# Functional phytochemicals in tomatoes: biosynthesis, gene regulation, and human health implications

**DOI:** 10.3389/fpls.2025.1662388

**Published:** 2025-11-17

**Authors:** Essam ElShamey, Yawen Zeng, Yumei Ding, Jiazhen Yang

**Affiliations:** 1 Biotechnology and Germplasm Resources Institute, Yunnan Academy of Agricultural Sciences, Kunming, China; 2 Rice Research Department, Field Crops Research Institute, Agricultural Research Center, Giza, Egypt; 3 College of Food Science and Technology, Yunnan Agricultural University, Kunming, China

**Keywords:** tomato, functional components, biosynthesis pathways, gene regulation, health benefits, carotenoids, flavonoids

## Abstract

The nutritional and health-promoting properties of tomatoes (Solanum lycopersicum), a highly significant crop, are attributed to their abundance of beneficial components, such as flavonoids, phenolic compounds, and carotenoids (including lycopene and β-carotene). The occurrence of these bioactive molecules is influenced by genetic, environmental, and agronomic factors, with ripening playing a critical role in their accumulation. This abstract delves into the molecular machinery controlling phytochemical accumulation, with a specific focus on the regulation of lycopene biosynthesis. The RIPENING-INHIBITOR (RIN) transcription factor, a master regulator of fruit maturation, exerts direct control over lycopene accumulation by binding to the promoters of critical biosynthetic genes. RIN directly activates the expression of PHYTENE SYNTHASE 1 (PSY1), the key rate limiting enzyme committing metabolic flux to the carotenoid pathway, and PDS, encoding phytocene desaturase, thereby orchestrating the massive lycopene synthesis characteristic of the ripening transition. Strategies for the biofortification of tomato fruits have leveraged this understanding through targeted genetic manipulation. Overexpression of key enzymes, such as the bacterial CrtB (phytoene synthase) or manipulation of the endogenous PSY1, has successfully enhanced lycopene flux. More profoundly, the manipulation of transcription factors offers a powerful multi-gene approach. For instance, the overexpression of fruit-specific promoters driving RIN or other regulators like HYR (High Pigment) can simultaneously improve the entire pathway, leading to substantial increases in lycopene content. Flavonoids and phenolic compounds are produced by the phenylpropanoid pathway, which is regulated by enzymes such as chalcone synthase (CHS) and phenylalanine ammonialyase (PAL). Gene regulation of these pathways involves a complex interplay of transcription factors (e.g., RIN, NOR, and HY5) and phytohormones (e.g., ethylene and abscisic acid), which modulate expression patterns during fruit development and stress responses. Phytochemical levels are also significantly influenced by environmental factors; for instance, optimal lycopene synthesis occurs at 20-25 °C, while higher temperatures above 30 °C inhibit lycopene accumulation and promote beta-carotene synthesis, a shift mediated by the temperature-sensitive expression of key genes, including those regulated by RIN. Naturally occurring or induced mutations in genes such as DET1 and HP2, which are negative regulators of light signal transduction, result in high pigment phenotypes with dramatically increased lycopene and flavonoid content. The successful application of metabolic engineering and transcription factor manipulation for biofortification holds immense promises for developing next-generation tomato cultivars with amplified health-promoting properties, directly linking agricultural science to improved human health outcomes through the mitigation of chronic diseases like cancer and cardiovascular disorders.

## 1 Introduction

The tomato (*Solanum lycopersicum*) is one of the most popular and nutrient-dense vegetables in the world (Kumar et al., 2020). In addition to their many culinary uses, tomatoes are a great source of health promoting bioactive chemicals (Pinela et al., 2016). Flavonoids (like quercetin and kaempferol), phenolic acids (like chlorogenic acid), carotenoids (like lycopene and β-carotene), glycoalkaloids (like α-tomatine), and vitamins (like C and E) are some examples of these bioactive components useful substances (Szabo et al., 2025; Dūma et al., 2018). These compounds exhibit antioxidant, anti-inflammatory, anticancer, and cardioprotective properties, making tomatoes a functional food with significant health benefits. The occurrence of these phytochemicals varies depending on genetic factors, environmental conditions, and postharvest handling (Tiwari and Cummins, 2013). Their biosynthesis is regulated by complex metabolic pathways involving key enzymes and transcription factors (Li et al., 2025). Understanding the genetic and molecular mechanisms behind their production can help in developing biofortified tomato varieties with enhanced nutritional value (Meng et al., 2022; Ofori et al., 2022). This article provides an in-depth exploration of the functional components in tomatoes, covering their; occurrence distribution in different tomato tissues and varieties, biosynthesis pathways key enzymatic steps in the production of carotenoids, flavonoids, and other metabolites, gene regulation transcriptional and post-transcriptional control of biosynthetic genes, and health benefits evidence-based roles in disease prevention and health promotion. By elucidating these aspects, we aim to highlight the importance of tomatoes as a dietary source of bioactive compounds and discuss potential strategies for enhancing their nutritional quality through breeding and biotechnology.

**Figure 1:**
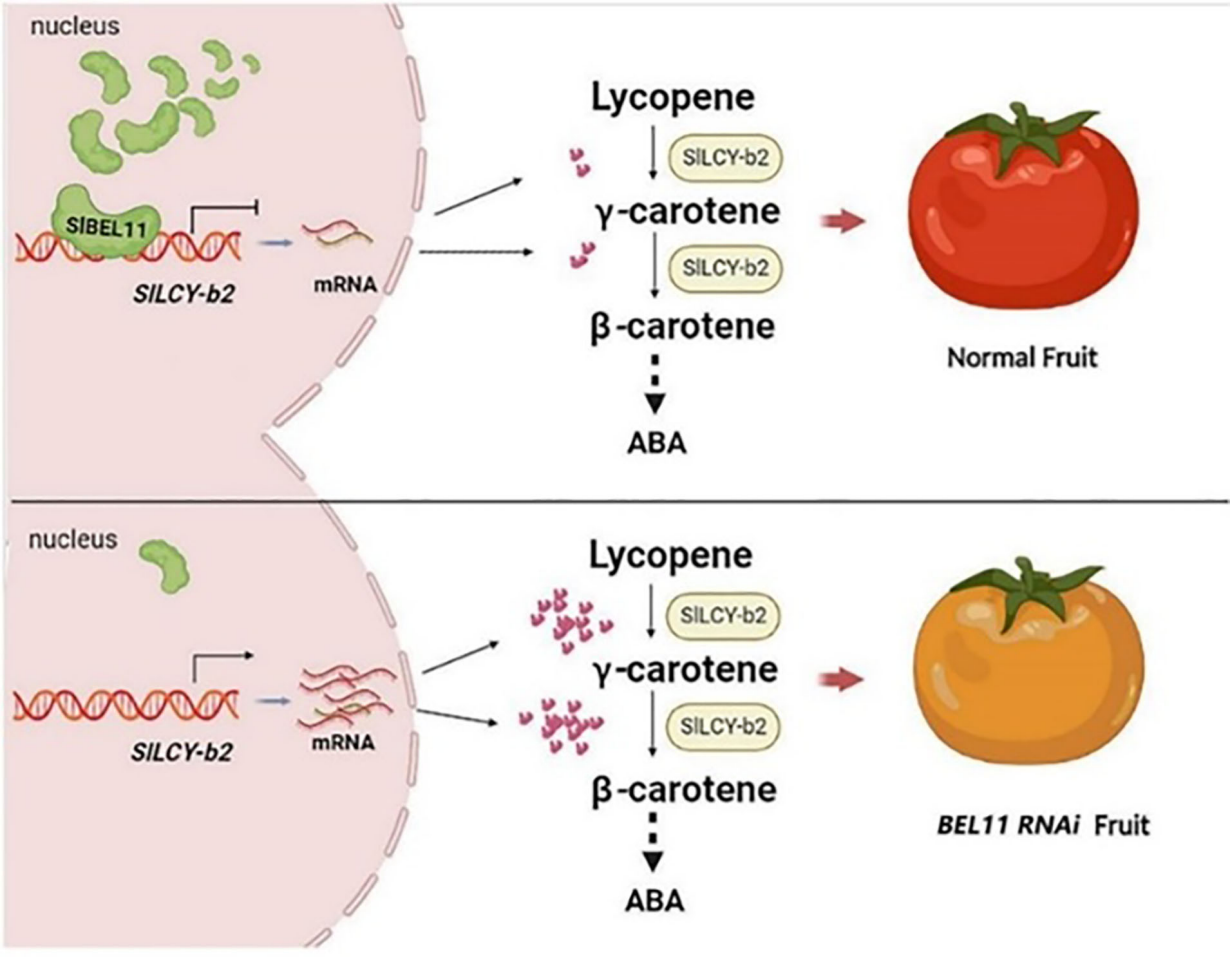
*SlBEL11* and its roles in biosynthesis carotenoid in tomato (He et al., 2022).

Occurrence of functional components, tomatoes accumulate various phytochemicals in different tissues; such as carotenoids (lycopene, β-carotene, lutein) are predominantly found in the ripened fruit (Chaudhary et al., 2018; Wang et al., 2023b), with lycopene being the most abundant. Flavonoids (naringenin, rutin, quercetin) are concentrated in the peel and outer pericarp. Phenolic acids (chlorogenic acid, caffeic acid) are distributed throughout the fruit (Suleria et al., 2020). Glycoalkaloids (α-tomatine) are more abundant in green tomatoes and leaves (Kozukue et al., 2023). Factors such as cultivar type, ripening stage, light exposure, and agronomic practices influence their concentrations (Cervantes et al., 2019). Biosynthesis pathways of key phytochemicals; carotenoid biosynthesis derived from the methylerythritol phosphate (MEP) pathway (Saadullah et al., 2025), leading to geranylgeranyl pyrophosphate (GGPP) (Ezquerro, 2022). Phytoene synthase (PSY) catalysis the first committed step, forming phytoene (Zhou et al., 2022), the subsequent desaturation and isomerization reactions produce lycopene, which can be cyclized into β-carotene (Heymann et al., 2015), **Figure 1** explain roles of transcription factor (SlBEL11) in biosynthesis of carotenoids. Flavonoid biosynthesis originates from the phenylpropanoid pathway, producing precursors like p-coumaroyl-CoA. Chalcone isomerase (CHI) and chalcone synthase (CHS) lead to naringenin chalcone, a precursor for various flavonoids (Tong et al., 2021; Saltzman, 2023; Waki et al., 2020). For glycoalkaloid biosynthesis derived from cholesterol, undergoing glycosylation to form α-tomatine, which decreases during fruit ripening. Gene regulation of biosynthetic pathways through transcription factors (TFs) such as RIN (Ripening Inhibitor), HY5 (Elongated Hypocotyl 5), and MYB regulators control carotenoid and flavonoid production (Liu et al., 2023; Xie et al., 2024). Epigenetic modifications (histone acetylation, DNA methylation) induce gene expression during ripening and the environmental signals (light, temperature) modulates biosynthetic gene activity via photoreceptors like phytochromes (Bianchetti et al., 2022; Li et al., 2022; Anwar et al., 2021).

Health benefits of tomato bioactive compounds (lycopene, flavonoids, vitamin C, and α-tomatine) can reduces oxidative stress, lowers cardiovascular disease risk, and exhibit anticancer properties (especially prostate cancer) (Pinela et al., 2016; Friedman, 2013; Collins et al., 2022). Also, improve endothelial function and possess anti-inflammatory effects, enhances immune function and collagen synthesis, and shows antimicrobial and cholesterol lowering effects (Woźniak et al., 2023). Tomatoes are a powerhouse of bioactive compounds with significant health promoting properties. While the targeted manipulation of gene regulation holds immense promise for enhancing functional phytochemicals in tomatoes, the approach is not without its significant current limitations. Scientifically, a primary concern remains the potential for off-target effects, where gene-editing tools like CRISPR-Cas9 could inadvertently alter unintended sections of the genome, potentially disrupting other vital metabolic pathways or plant functions, with consequences that are difficult to fully predict. Beyond the laboratory, consumer acceptance and complex regulatory landscapes present formidable hurdles. Widespread public skepticism, particularly regarding “GMO” technologies, and stringent, varying global regulations could severely limit the commercial viability and market access of these nutritionally enhanced tomatoes. Therefore, addressing these dual challenges of technical precision and societal trust is as crucial as the scientific breakthrough itself for the successful application of gene regulation in creating the next generation of functional foods. Advances in genomics and metabolic engineering offer opportunities to enhance these functional components, paving the way for improved dietary strategies and functional food development. Further research into gene regulation and bioavailability will maximize their therapeutic potential.

## 2 Occurrence of functional components in tomato

Tomatoes (*Solanum lycopersicum*) have a wide range of bioactive chemicals with important nutritional and health benefits. Carotenoids (including lycopene and β-carotene), phenolic acids, flavonoids, vitamins C and E, and glycoalkaloids are responsible for the fruit’s anti-inflammatory, anti-cancer, and antioxidant qualities (**Figure 1**). Genetic cultivar, ripening stage, and agronomic conditions all influence the composition and concentration of these bioactive compounds, which are highly variable. For instance, lycopene, the predominant carotenoid responsible for tomatoes’ red colour, accumulates predominantly during the later stages of ripening, while certain flavonoids and chlorogenic acid levels may peak earlier. Environmental factors, including light exposure, temperature, soil quality, and water availability, further modulate phytochemical profiles, with organic cultivation and stress conditions (e.g., drought or salinity) often enhancing secondary metabolite production. Additionally, postharvest handling and processing methods (e.g., thermal treatment) can alter bioavailability and bioactivity. Understanding these dynamics is crucial for optimizing tomato production to maximize health benefits and for developing functional foods or nutraceuticals. The concentration of these phytochemicals varies significantly between green and ripening stages, influenced by biochemical and enzymatic changes during maturation as shown in **Table 1**. Lycopene, the predominant carotenoid in ripe tomatoes, increases dramatically during ripening due to the upregulation of lycopene biosynthesis pathways (Bramley, 2002; Zhu et al., 2022), while β-carotene levels may show a more gradual rise (Kozukue and Friedman, 2003; Kapoor et al., 2022). Phenolic acids and flavonoids, which are key antioxidants, often peak at intermediate ripening stages, as their synthesis is modulated by both developmental cues and environmental factors (Valero and Serrano, 2013). Vitamin C (ascorbic acid) tends to accumulate progressively with ripening (Fenech et al., 2019), whereas vitamin E (tocopherols) may exhibit a more stable or slightly declining trend (Galli and Azzi, 2010). Glycoalkaloids, such as α-tomatine, are typically higher in green tomatoes and decline as the fruit matures, meaning that glycoalkaloids play a role like a defense mechanism shift (Friedman, 2002; Faria-Silva et al., 2022). This dynamic profile of bioactive compounds highlights the importance of harvest timing in optimizing nutritional quality. Understanding these metabolic changes provides insights into breeding strategies and postharvest practices aimed at enhancing the health benefits of tomatoes for human consumption.

**Table 1:** Summarizing the concentration changes of key bioactive compounds in green (unripe) and ripening (red) tomatoes (*Solanum lycopersicum*).

Compound	Green tomato	Ripening tomato (red)	Change during ripening	Ref.
Carotenoids				
Lycopene	Low	High	Dramatic increase	(Bramley, 2002; Kapoor et al., 2022)
β-Carotene	Low	Moderate	Gradual increase	(Kozukue and Friedman, 2003,; Kapoor et al., 2022)
Phenolic Acids	High	Moderate to Low	Slight decrease	(Valero and Serrano, 2013; Gupta et al., 2022)
Flavonoids	High	Moderate	Decreases slightly	(Fenech et al., 2019)
Vitamins				
Vitamin C	Moderate	High	Increases	(Fenech et al., 2019; Mellidou and Kanellis, 2023)
Vitamin E (α-tocopherol)	Low	Moderate	Slight increase	(Galli and Azzi, 2010)
Glycoalkaloids	High (e.g., α-tomatine)	Low	Decreases significantly	(Liu et al., 2023)

### 2.1 Carotenoids

Carotenoids are a class of bioactive compounds widely recognized for their antioxidant properties, with tomatoes (*Solanum lycopersicum*) being one of the richest dietary sources (León-García et al., 2017). The major carotenoids in tomatoes include lycopene, β-carotene, lutein, and zeaxanthin, each contributing to the fruit’s vibrant color and nutritional value (Ilahy et al., 2018). Among these, lycopene stands out as the most abundant, accounting for approximately 80–90% of total carotenoids, and is renowned for its potent antioxidant and anti-inflammatory activities (Khan et al., 2021). Epidemiological and clinical studies have linked lycopene consumption to a reduced risk of chronic diseases, including cardiovascular disorders, certain cancers, and age-related macular degeneration (Abir et al., 2023). The biosynthesis of carotenoids in tomatoes is influenced by genetic, environmental, and agronomic factors, such as cultivar type, ripening stage, light exposure, and postharvest handling (Saini and Keum, 2018; Nie et al., 2024). Recent advances in metabolic engineering and breeding strategies have aimed to enhance carotenoid content, particularly lycopene and β-carotene, to improve nutritional quality. Additionally, the bioavailability of tomato carotenoids is affected by food processing methods; thermal treatment and lipid co-consumption have been shown to increase their absorption in the human digestive system (Yan et al., 2024; Rocha et al., 2023). Despite their health benefits, carotenoid stability is a challenge due to susceptibility to oxidative degradation. Encapsulation techniques and antioxidant-rich dietary matrices are being explored to preserve their bioactivity (Borah et al., 2023; Saini et al., 2022). Future research should focus on optimizing carotenoid retention in processed tomato products, elucidating their molecular mechanisms in disease prevention, and developing biofortified tomato varieties to address global nutritional deficiencies.

### 2.2 Polyphenols

Polyphenols are a diverse group of bioactive compounds in tomatoes (*Solanum lycopersicum*), these secondary metabolites, including flavonoids (e.g., quercetin, kaempferol, and naringenin) and phenolic acids (e.g., chlorogenic and caffeic acids), contribute significantly to the fruit’s nutritional and functional value (Wang et al., 2023b; Berni et al., 2018). The polyphenolic composition in tomatoes like carotenoids varies depending on genetic factors, ripening stage, agronomic practices, and post-harvest conditions (Tilahun et al., 2017). These chemicals serve an important role in alleviating oxidative stress by scavenging free radicals and altering cellular signaling pathways, hence lowering the risk of chronic diseases such as cardiovascular disorders, cancer, and diabetes. Additionally, polyphenols in tomatoes exhibit antimicrobial and anti-proliferative activities, further enhancing their potential as nutraceuticals (D’Angelo, 2021; Mutalib et al., 2023). Recent advances in metabolomics and biofortification strategies have enabled the enhancement of polyphenol content in tomatoes, offering improved dietary sources for health benefits. However, bioavailability and metabolism of these compounds in humans remain critical areas of research to fully exploit their therapeutic potential.

### 2.3 Vitamins

Tomatoes (*Solanum lycopersicum*) are particularly rich in vitamins, which significantly increases their nutritional worth and health promoting properties (Pinela et al., 2016; Faraone et al., 2021). Among the most notable vitamins in tomatoes are vitamin C (ascorbic acid), vitamin A (primarily as β-carotene, a provitamin A carotenoid), vitamin E (α-tocopherol), and several B-complex vitamins, including folate (B9), pyridoxine (B6), and niacin (B3) (Amr and Raie, 2022; Ofoedu et al., 2021). These vitamins function as potent antioxidants, coenzymes, and regulators of metabolic processes, playing crucial roles in human health. Reactive oxygen species (ROS) are scavenged by vitamin C, a water-soluble antioxidant, enhances immune function, and aids in collagen synthesis (Khadim and Al-Fartusie, 2021). Lipophilic vitamins, such as β-carotene (a precursor to retinol) and α-tocopherol, protect cellular membranes from oxidative damage and support vision, skin health, and immune responses (Khadim and Al-Fartusie, 2021; Carazo et al., 2021). Additionally, B vitamins in tomatoes contribute to energy metabolism, DNA synthesis, and neurological function. The bioavailability of these vitamins in tomatoes is influenced by factors such as ripening stage, cultivar differences, postharvest handling, and processing methods (Arah et al., 2015). Thermal processing, for instance, may degrade heat-labile vitamins like vitamin C but can increase the bioavailability of lipid-soluble vitamins by breaking down cell walls. Recent research highlights the synergistic interactions between tomato vitamins and other phytochemicals (e.g., lycopene, flavonoids), amplifying their antioxidant and anti-inflammatory effects. Given their essential roles in disease prevention such as reducing the risk of cardiovascular diseases, cancer, and age-related degeneration tomato derived vitamins represent key functional food components. Further studies on biofortification, optimized processing techniques, and the mechanistic pathways of these vitamins could enhance their therapeutic and nutraceutical applications.

### 2.4 Glycoalkaloids

The class of nitrogen-containing secondary metabolites known as glycoalkaloids is mostly present in plants of the Solanaceae family, which includes tomatoes (*Solanum lycopersicum*) (Zhao et al., 2021). Because of their antibacterial, antifungal, and insecticidal qualities, these bioactive substances like α-tomatine and dehydrotomatine are essential to plant defense mechanisms against diseases and pests (Liu et al., 2023). Recent studies have highlighted their potential pharmacological benefits, including anticancer, anti-inflammatory, and cholesterol-lowering effects, making them promising candidates for nutraceutical and therapeutic applications. However, glycoalkaloids exhibit a dual nature, as excessive consumption may lead to toxic effects such as gastro intestinal disturbances and neurotoxicity, necessitating careful consideration of their dosage and bioavailability (Ahamad et al., 2022). The biosynthesis of glycoalkaloids in tomatoes is influenced by genetic, environmental, and postharvest factors, with ripening stages significantly affecting their concentration (Zhao et al., 2023). Advanced extraction and analytical techniques, such as HPLC-MS (ElShamey et al., 2021) and NMR (ElShamey et al., 2021), have enabled precise quantification and structural characterization, facilitating research into their bioactivity and safety profiles. This review comprehensively examines the biochemical properties, biological functions, health implications, and potential applications of tomato glycoalkaloids, while addressing challenges related to their toxicity and regulatory aspects.

### 2.5 Other bioactive compounds

Tomatoes (*Solanum lycopersicum*) are a rich source of diverse bioactive compounds beyond the well-studied carotenoids, vitamins, polyphenols, and glycoalkaloids. These lesser explored phytochemicals exhibit significant biological activities, contributing to the health promoting properties of tomatoes. Among them, terpenes (such as mono and sesquiterpenes) contribute to aroma and possess antimicrobial and anti-inflammatory effects. Phytosterols (e.g., β-sitosterol and stigmasterol) demonstrate cholesterol lowering potential and may modulate cardiovascular health (Kumar et al., 2022; Lobo et al., 2018). Fatty acid derivatives, including oxylipins, play roles in plant defense and exhibit anti-inflammatory and antioxidant properties in humans (Savchenko et al., 2022). Additionally, alkaloids like tomatidine (an aglycone of α-tomatine) show emerging anticancer and antimicrobial activities (Friedman, 2013; Faria-Silva et al., 2022). Flavonoid glycosides (distinct from free polyphenols) enhance bioavailability and exert antioxidant effects, Plants package certain healthy compounds (flavonoids) with a sugar molecule. This package job makes them more easily absorbed into your body than their unpackaged versions. Once absorbed, they help protect your cells from damage. Furthermore, nucleosides and nucleotides in tomatoes may influence cellular metabolism and immune function (Witte and Herde, 2024). The presence of sulfur-containing compounds, such as glutathione derivatives, contributes to redox regulation and detoxification processes. These under investigated compounds, though present in smaller quantities, may synergize with major phytochemicals to enhance the nutraceutical value of tomatoes. To clarify their processes, bioavailability, and possible health advantages in disease prevention and the creation of functional foods, more investigation is required.

## 3 Agricultural practices and phytochemical concentrations

Recent research has demonstrated that tailored agricultural practices can significantly enhance the biosynthesis and accumulation of these valuable compounds as shown in **Table 2**. This review explores key cultivation strategies that optimize light exposure, precision nutrient management, and controlled stress induction that can be employed to elevate phytochemical concentrations in tomato fruits. Optimized light exposure plays a critical role in modulating secondary metabolite production (Zhang et al., 2021). Adjusting light quality (e.g., red, blue, and UV-B spectra), intensity, and photoperiod can stimulate the phenylpropanoid and carotenoid pathways, leading to increased synthesis of flavonoids, anthocyanins, and lycopene (Wang et al., 2025). For instance, supplemental blue light has been shown to enhance antioxidant capacity, while UV-B exposure can trigger defense related phytochemical accumulation (Bhattarai et al., 2025). Precision nutrient management, particularly the modulation of macronutrients (N, P, K) and micronutrients (Mg, Zn, Se), influences enzymatic activities involved in phytochemical biosynthesis (Tariq et al., 2023). Reduced nitrogen levels, coupled with balanced potassium and phosphorus, have been linked to higher phenolic and carotenoid content. Additionally, biofortification with selenium and zinc can further augment antioxidant properties without compromising yield.

**Table 2:** Summarizing agricultural practices that can enhance the biosynthesis and accumulation of valuable compounds in green and ripening tomatoes (*Solanum lycopersicum*).

Agricultural practice	Effect on green tomatoes	Effect on ripening tomatoes	Key compounds enhanced	Mechanism/notes	Ref.
Optimized Light Exposure	Increases chlorophyll and phenolic content	Enhances lycopene and carotenoid synthesis	Phenolics, flavonoids, lycopene, β-carotene	UV-B and red light stimulate antioxidant pathways	(Cervantes et al., 2019)
Water Stress (Moderate)	May increase phenolic acids	Boosts lycopene and sugar content	Lycopene, phenolics, sugars	Controlled drought stress enhances secondary metabolites	(Rouphael et al., 2012)
Nutrient Management (High K, Low N)	Improves early growth and chlorophyll	Enhances color development and antioxidants	Lycopene, carotenoids, vitamin C	Potassium promotes ripening; excess nitrogen delays it	(Rouphael et al., 2012)
Organic Fertilization (Compost, Biofertilizers)	Increases phenolic and flavonoid content	Improves lycopene and antioxidant capacity	Flavonoids, lycopene, vitamin E	Microbes enhance nutrient uptake and stress resilience	(Rouphael et al., 2012)
Ethephon or Ethylene Application	Accelerates color change (if applied late)	Significantly boosts lycopene synthesis	Lycopene, carotenoids	Ethylene triggers ripening-related pathways	(Kowsalya et al., 2025)
Temperature Modulation (Warm days, cool nights)	Slows degradation of chlorophyll	Promotes lycopene accumulation	Lycopene, β-carotene	Optimal temps (~24 °C day, ~18 °C night) favor pigment synthesis	(Ferrante and Mariani, 2018)
Salinity Stress (Moderate)	May increase osmoprotectants (e.g., proline)	Enhances phenolic and lycopene content	Phenolics, lycopene, vitamin C	Mild salt stress induces antioxidant responses	(Tariq et al., 2023)
Use of Biostimulants (Seaweed extracts, humic acids)	Improves early growth and stress tolerance	Enhances pigment and nutrient content	Lycopene, flavonoids, vitamin C	Stimulates plant defense and metabolic activity	(Vardanian et al., 2025)
Harvest Time (Breaker stage vs. Mature green)	Higher chlorogenic acid if harvested early	Optimal lycopene if vine-ripened	Lycopene, flavonoids, vitamin C	Longer vine retention increases phytonutrients	(Vardanian et al., 2025)
Mulching (Plastic or Organic)	Stabilizes root zone, improves early growth	Enhances sugar and antioxidant levels	Lycopene, phenolics, sugars	Regulates soil temp/moisture for better nutrient uptake	(La Spada et al., 2024)

Controlled stress induction through moderate drought, salinity, or biotic elicitors (e.g., jasmonic acid, chitosan) activates plant defense mechanisms, resulting in the upregulation of secondary metabolites. Abiotic stresses such as regulated deficit irrigation and saline conditions have been found to boost lycopene and tocopherol levels, while elicitors can enhance the production of polyphenols and glycoalkaloids. Integrating these agronomic approaches offers a sustainable strategy to enhance the nutraceutical value of tomatoes while maintaining crop productivity. Future research should focus on genotype-specific responses and the economic feasibility of large-scale implementation to maximize phytochemical yields for functional foods and pharmaceutical applications.

## 4 Biosynthesis pathways of key functional components

### 4.1 Carotenoid biosynthesis

**Figure 2:**
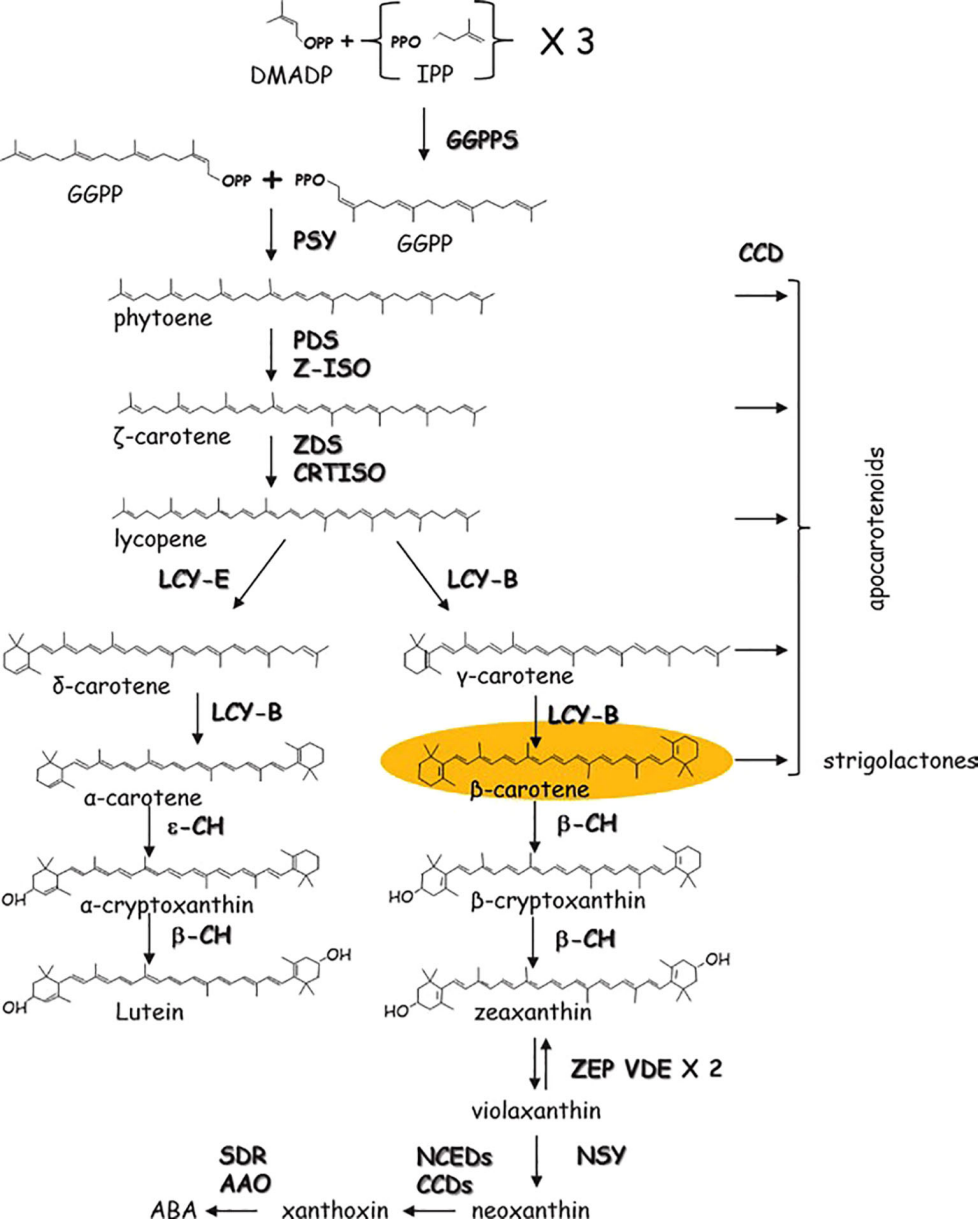
Schematic biosynthesis pathways for carotenoids in tomato fruits.

The biosynthesis of carotenoids in tomato fruits represents a brilliantly orchestrated biochemical pathway, transitioning chloroplasts into chromoplasts and painting the ripening fruit with characteristic red and orange hues. This process is governed by a precise genetic and enzymatic framework. The pathway initiates with the condensation of geranylgeranyl pyrophosphate (GGPP) by phytoene synthase (PSY1), the first and major rate-limiting enzyme encoded by the *PSY1* gene, which is exclusively and highly expressed during fruit ripening. The colorless phytoene is then progressively desaturated and isomerized by a series of enzymes, including phytoene desaturase (PDS), ζ-carotene desaturase (ZDS), and the carotenoid isomerase (CRTISO), to form the red pigment, lycopene. This lycopene accumulation is the hallmark of ripe tomato fruit. The cyclization of lycopene, catalyzed by lycopene β-cyclase (LCY-B) and lycopene ϵ-cyclase (LCY-E), branches the pathway towards the production of β-carotene (provitamin A) and lutein. The critical shift from chloroplastic to chromoplastic carotenoid accumulation is tightly regulated by ripening transcription factors, most notably the RIN (RIPENING INHIBITOR), NOR (NON-RIPENING), and TAGL1 proteins, which directly activate key genes like *PSY1*. The production of the basic C5 isoprenoid units by the methylerythritol 4-phosphate (MEP) pathway is the first in a series of enzymatic mechanisms that tightly control the biosynthesis of carotenoids in tomatoes, which primarily occurs in the chromoplasts. The first committed carotenoid precursor, phytoene, is created when phytoene synthase (PSY) condenses these units. A number of desaturation, isomerization, and cyclization processes are catalyzed by important enzymes such as phytoene desaturase (PDS), ζ-carotene desaturase (ZDS), carotenoid isomerase (CRTISO), and lycopene β- and ϵ-cyclases (LCYB, LCYE) as shown in **Figure 2** (Llorente et al., 2016). The regulation of these pathways is influenced by genetic factors (e.g., transcription factors *RIN*, *NOR*, and *HYR*), environmental conditions (light, temperature), and hormonal signals (ethylene, abscisic acid).

Recent advances in molecular biology have provided powerful tools to dissect and manipulate this pathway with unprecedented precision. While traditional Mutagenesis and QTL (Quantitative Trait Loci) mapping identified foundational genes like old-gold (responsible for β-carotene accumulation), modern techniques have revolutionized our capabilities; CRISPR-Cas9 genome editing, this has become the technique of choice for targeted metabolic engineering. By knocking out specific genes, scientists can precisely redirect metabolic flux. For instance, knocking out LCY-B prevents the conversion of lycopene to β-carotene, resulting in tomatoes with significantly enhanced lycopene content. Conversely, simultaneous editing can create novel profiles, such as high-β-carotene fruits. Transcriptomics (RNA-Seq), this allows for the comprehensive profiling of gene expression throughout fruit development and in response to various conditions. By comparing transcriptomes of different tomato varieties or ripening stages, researchers can identify novel genes and regulatory networks involved in carotenoid control, beyond the well-established pathway. Finally, metabolomics which coupled with transcriptomics, metabolomics provides a complete snapshot of the metabolic profile. This systems biology approach helps in understanding the complex interactions between the carotenoid pathway and other metabolic networks, revealing how engineering one pathway might affect others.

In summary, the journey from a green to a red tomato is a vivid demonstration of precise genetic control over a defined enzymatic pathway, with PSY1 and LCY-B acting as critical gatekeepers. The deployment of sophisticated molecular techniques, particularly CRISPR-Cas9, has transitioned research from mere observation to direct, precise engineering. These tools not only deepen our fundamental understanding of plant metabolism but also hold immense promises for biofortification, allowing us to design tomato fruits with optimized nutritional value, enhanced visual appeal, and improved health benefits to meet global dietary needs.

### 4.2 Flavonoid biosynthesis

**Figure 3:**
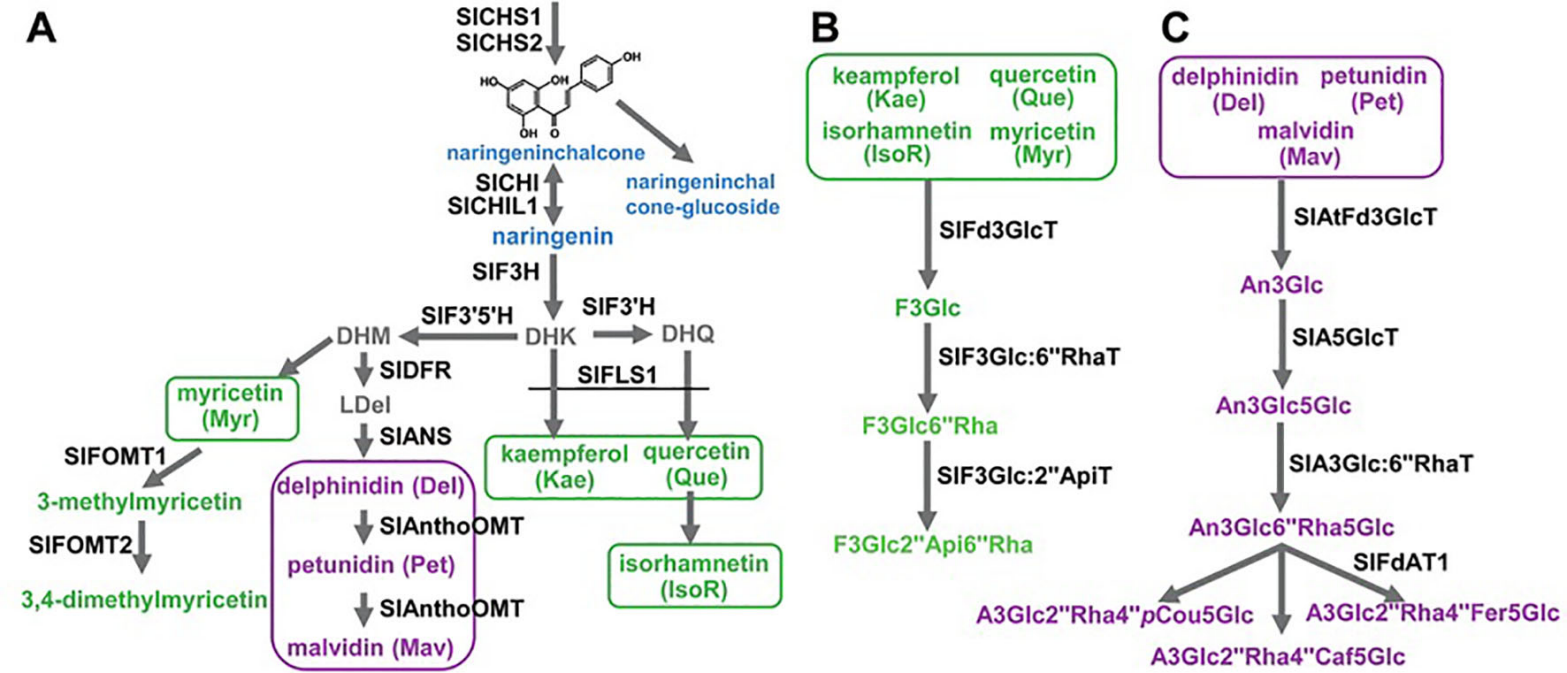
Schematic biosynthesis pathways for flavonoids in tomato fruits.

The biosynthesis of flavonoids in tomato fruits is a meticulously regulated biochemical pathway, resulting in a spectrum of beneficial compounds ranging from colorless flavanones to brightly colored anthocyanins. This pathway is orchestrated by a core set of structural genes encoding enzymes that sequentially modify the basic phenylpropanoid backbone. The journey begins with PAL (Phenylalanine Ammonia-Lyase), which channels primary metabolism into the pathway, and proceeds through key enzymes like CHS (Chalcone Synthase), CHI (Chalcone Isomerase), and F3H (Flavanone 3-Hydroxylase) to form the central intermediate, dihydrokaempferol. The pathway then diverges, guided by the action of DFR (Dihydroflavonol 4-Reductase), ANS (Anthocyanidin Synthase), and various Glycosyltransferases and Methyltransferases, to produce the final array of pigments and compounds, such as the red anthocyanins in the fruit peel or flavonols like quercetin and kaempferol glycosides. Crucially, the spatial and temporal expression of these structural genes is governed by a complex of transcriptional regulators, primarily from the R2R3-MYB, bHLH (basic Helix-Loop-Helix), and WDR (WD-repeat) protein families (Li, 2014; Gao et al., 2018). In tomato, the activation of anthocyanin biosynthesis, for instance, is often dependent on the expression of specific MYB transcription factors (e.g., ANT1, AN2-like), which interact with bHLH partners (e.g., AN1) to form the MBW complex that activates the promoters of late biosynthetic genes like DFR and ANS as shown in **Figure 3** (Verhoeyen et al., 2002; Schijlen et al., 2006).

Recent advances in molecular biology have revolutionized our ability to dissect and manipulate this pathway. CRISPR-Cas9 gene editing has been instrumental, moving beyond correlation to direct causation by knocking out specific genes (e.g., *CHS1*, *DFR*) to confirm their function and create tomatoes with altered flavonoid profiles. Conversely, the targeted activation of key transcription factors like SIAN2 or SIMYB75 using CRISPR activation (CRISPRa) systems or traditional transgenesis has successfully engineered tomatoes with dramatically enhanced anthocyanin accumulation, turning the fruit purple and boosting its antioxidant capacity. Furthermore, RNA interference (RNAi) has been used to silence specific genes, fine-tuning the pathway to reduce undesirable compounds or shunt flux towards preferred flavonoids. Beyond single-gene manipulation, multi-omics approaches integrating genomics, transcriptomics, metabolomics, and proteomics have provided a systems level understanding. By analyzing the entire pathway simultaneously, researchers can identify all players involved, uncover novel regulatory nodes, and understand how environmental factors influence flavonoid output. Virus-Induced Gene Silencing (VIGS) remains a rapid, powerful tool for transiently knocking down gene expression in tomato fruits, allowing for high-throughput functional screening of candidate genes without the need for stable transformation. In summary, the flavonoid pathway in tomato is a well-defined genetic and enzymatic network. The synergy between classical biochemistry and cutting-edge molecular techniques particularly CRISPR-Cas9 and multi-omics integration has not only demystified the roles of key genes and enzymes but has also empowered breeders and biotechnologists to precisely engineer tomato fruits. This paves the way for developing next generation tomato varieties with enhanced nutritional value, improved stress resilience, and novel visual and health-promoting traits tailored to meet consumer and agricultural demands.

### 4.3 Vitamin C biosynthesis

**Figure 4:**
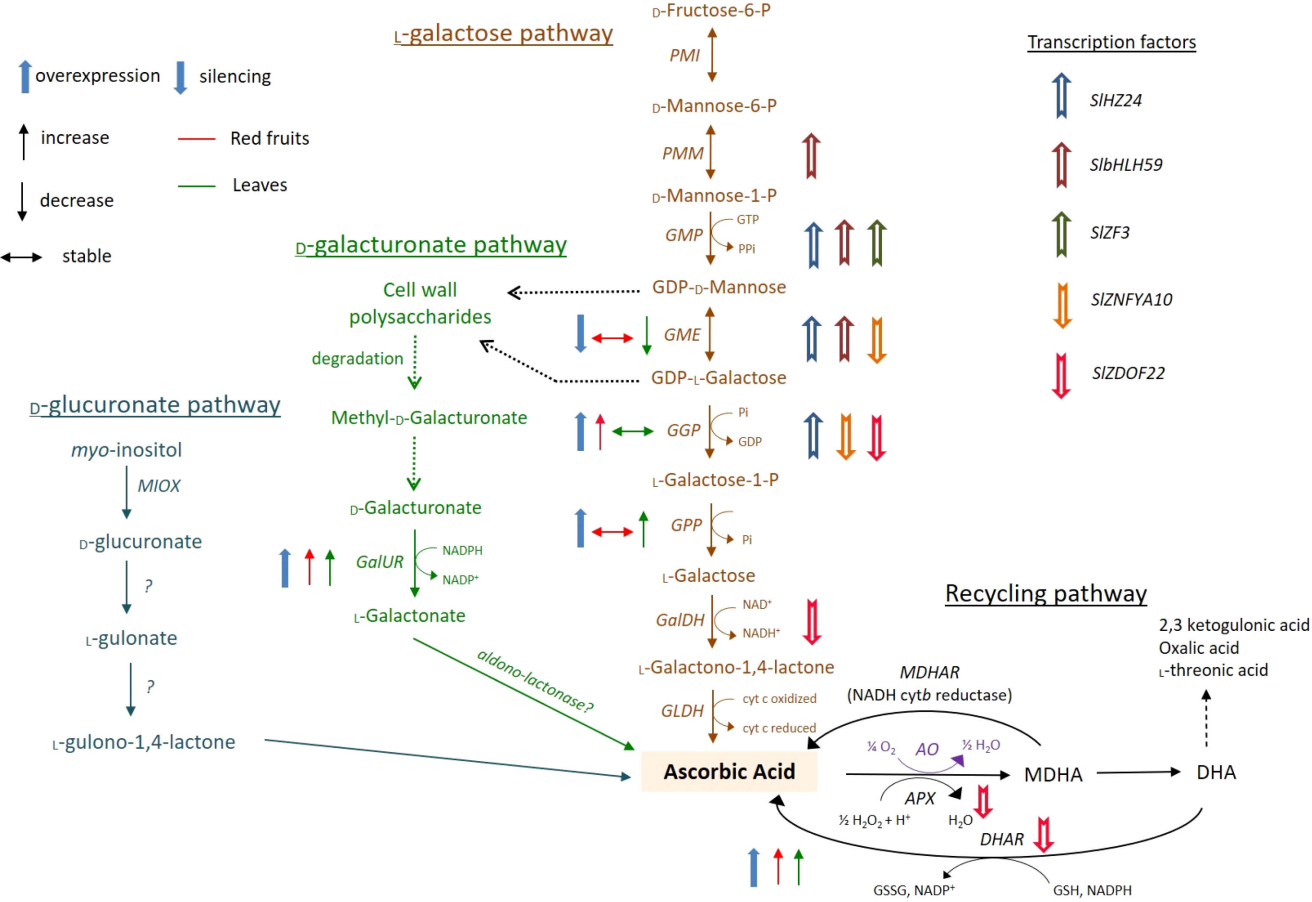
Schematic biosynthesis pathways for vitamin C in tomato fruits (Mellidou et al., 2021).

The biosynthesis of vitamin C (L-ascorbic acid, AsA) in tomato fruits is a complex and highly regulated process, primarily governed by the L-galactose pathway. This pathway represents the dominant route for *de novo* AsA production in plants, converting the nucleotide sugar GDP-D-mannose into L-ascorbic acid through a series of enzymatic steps. Key genes and enzymes central to this pathway in tomato include; GMP (GDP-D-mannose pyrophosphorylase) which encoded by genes like SIGMP, it catalyzes the conversion of D-mannose-1-P to GDP-D-mannose, serving as a critical early gatekeeper. GME (GDP-D-mannose-3’,5’-epimerase) which Encoded by SIGME, this enzyme performs a dual epimerization, producing GDP-L-galactose, a crucial precursor. GGP (GDP-L-galactose phosphorylase) which encoded by SIGGP, this is often considered the major flux-controlling step of the pathway. It catalyzes the conversion of GDP-L-galactose to L-galactose-1-P, and its expression and activity are strongly correlated with AsA accumulation in ripening fruits. GLDH (L-galactono-1,4-lactone dehydrogenase) the final enzyme in the pathway, encoded by SIGLDH, oxidizes L-galactono-1,4-lactone to AsA in the mitochondria, linking AsA biosynthesis to the mitochondrial electron transport chain. Beyond this main pathway, recycling via the ascorbate glutathione cycle, involving enzymes like MDHAR (Monodehydroascorbate Reductase) and DHAR (Dehydroascorbate Reductase), is crucial for maintaining the reduced pool of AsA by regenerating it from its oxidized forms, thus influencing the final vitamin C content in the ripe fruit as shown in **Figure 4** (Castro et al., 2023).

Recent advances in molecular biology have provided powerful tools to dissect and manipulate this biosynthetic network. Techniques such as CRISPR-Cas9-mediated gene editing have enabled the precise knockout of negative regulators or the fine-tuning of key biosynthetic genes to create tomato lines with enhanced AsA content without introducing foreign transgenes. Furthermore, Virus-Induced Gene Silencing (VIGS) has been instrumental as a rapid, high-throughput functional genomics tool to transiently knock down target genes in planta, allowing researchers to assess their role in AsA accumulation during fruit development. The integration of multi-omics approaches transcriptomics, proteomics, and metabolomics has been particularly transformative. By analyzing global changes in gene expression, protein levels, and metabolic fluxes, scientists can now identify not only the core biosynthetic genes but also novel transcription factors and regulatory networks that orchestrate AsA accumulation in coordination with fruit ripening and environmental responses. For instance, transcriptomic studies have revealed that several SIGGP and SIGME genes are upregulated during the breaker and ripening stages, coinciding with peak AsA levels. In summary, the vitamin C content in tomato fruits is a quantifiable trait determined by the concerted action of the L-galactose pathway genes and an efficient recycling system. The application of sophisticated molecular techniques like CRISPR-Cas9, VIGS, and multi-omics integration is rapidly moving the field from a descriptive understanding to a predictive and manipulative science. These tools are paving the way for the development of next-generation tomato cultivars with nutritionally enhanced levels of vitamin C, contributing to improved human health and agricultural value.

## 5 Gene regulation of functional components

Biosynthesis gene regulatory networks closely control the manufacture and accumulation of these metabolites, and transcription factors (TFs) are essential for modifying their expression, as **Table 3** illustrates. Key TFs, including R2R3-MYB, bHLH, WRKY, and AP2/ERF families, orchestrate the transcriptional activation or repression of structural genes involved in carotenoid (e.g., *PSY*, *LCY*, *CYCB*) and flavonoid pathways (e.g., *CHS*, *F3H*, *FLS*). Similarly, vitamin C (ascorbate) levels are influenced by TFs regulating genes in the L-galactose pathway (*GGP*, *GalDH*) and recycling enzymes (*MDHAR*, *DHAR*) (Liang and Li, 2023; Wu et al., 2022; Stanley and Yuan, 2019). Environmental stimuli, hormonal signals (such as ethylene and abscisic acid), and light-responsive TFs (e.g., HY5) further fine-tune these pathways, creating a dynamic interplay between genetic and external factors (Mo et al., 2021). Advances in omics technologies and CRISPR-based genome editing have unveiled novel TF-target interactions, providing opportunities for metabolic engineering to enhance tomato’s nutritional profile. This review synthesizes current knowledge on TF-mediated regulation of carotenoids, flavonoids, and vitamin C, highlighting potential strategies for biofortification and improved stress resilience in tomato cultivars.

**Table 3:** Summarizing gene regulation and transcriptional factors (TFs) involved in the biosynthesis of carotenoids, flavonoids, and vitamin C in tomato (*Solanum lycopersicum*).

Metabolite	Key biosynthetic genes	Transcriptional factors (TFs)	Regulatory mechanism	Ref.
Carotenoids (e.g., Lycopene, β-carotene)	PSY1, PDS, ZDS, LCY-B, CRTISO	RIN (Ripening Inhibitor)	Promotes carotenoid accumulation during fruit ripening by activating PSY1.	(Vrebalov et al., 2002; Fujisawa et al., 2013)
		NOR (non-ripening)	Regulates ripening-associated carotenoid biosynthesis.	(Giovannoni et al., 2017)
		HY5 (Elongated Hypocotyl 5)	Enhances light-mediated carotenoid production.	(Liu et al., 2018)
Flavonoids (e.g., Naringenin, Quercetin)	CHS, CHI, F3H, FLS SLMYB75	MYB12	Directly activates flavonoid pathway genes (CHS, CHI).	(Mathews et al., 2003)
		ANT1 (Anthocyanin 1)	Induces anthocyanin/flavonoid biosynthesis.	
			Positively regulates flavonol accumulation.	(Gong et al., 2021)
Vitamin C (Ascorbic acid)	GMP, GME, GLDH, GGP	SlHZ24	Negatively regulates ascorbate biosynthesis under high light.	(Castro et al., 2023; Liu et al., 2022)

### 5.1 Transcriptional regulation of carotenoids

**Figure 5:**
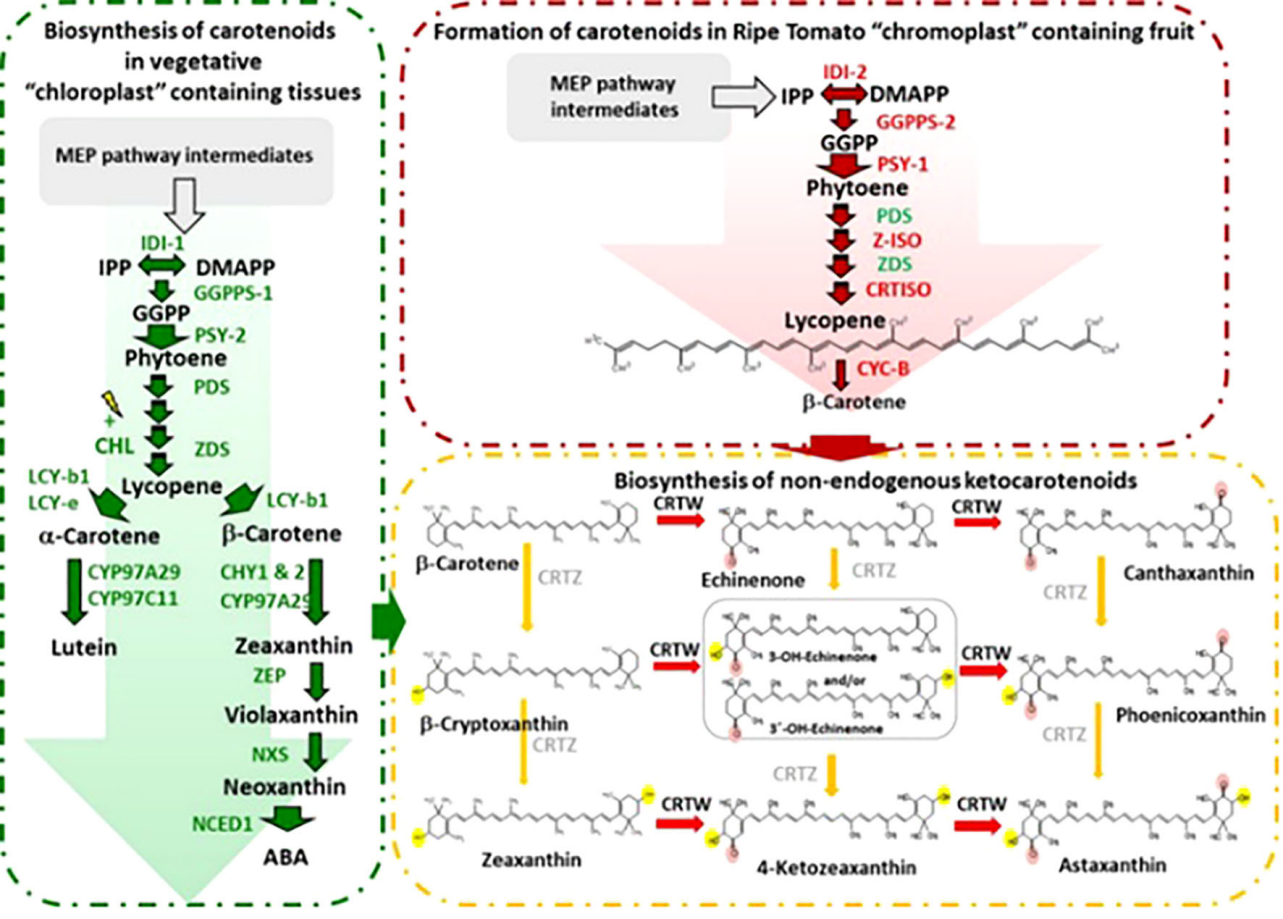
Biosynthesis of carotenoids in tomato chloroplast and chromoplast (Enfissi et al., 2019).

The transcriptional regulation of carotenoid biosynthesis in tomato (*Solanum lycopersicum*) involves a complex interaction of transcription factors (TFs), hormone signals, and environmental cues that modulate the expression of important biosynthetic genes (Wang et al., 2023a; Li et al., 2023). The phytoene synthase 1 (*PSY1*) gene initiates carotenoid production, while downstream enzymes like lycopene β-cyclase (*LCYB*) and β-carotene hydroxylase (*BCH*) impact lycopene and β-carotene accumulation. Key transcription factors, including ripening inhibitor (RIN), non-ripening (NOR), and colorless non-ripening (CNR), regulate carotenoid production during fruit ripening by binding to the promoters of carotenogenic genes. Additionally, phytochrome-interacting factors (PIFs) and HY5 (elongated hypocotyl 5) mediate light-dependent carotenoid regulation, while apetala2/ethylene-responsive factors (AP2/ERF) and mads-box proteins integrate ethylene and developmental signals (Filyushin et al., 2024; Sun et al., 2022). DNA methylation and histone acetylation are examples of epigenetic changes that make carotenoid gene expression even more precise. Understanding these regulatory networks provides insights into metabolic engineering strategies to enhance carotenoid content in tomatoes, improving both nutritional value and stress resilience, Biosynthesis of carotenoids in tomato chloroplast and chromoplast were shown in **Figure 5**.

### 5.2 Regulation of flavonoid biosynthesis

**Figure 6:**
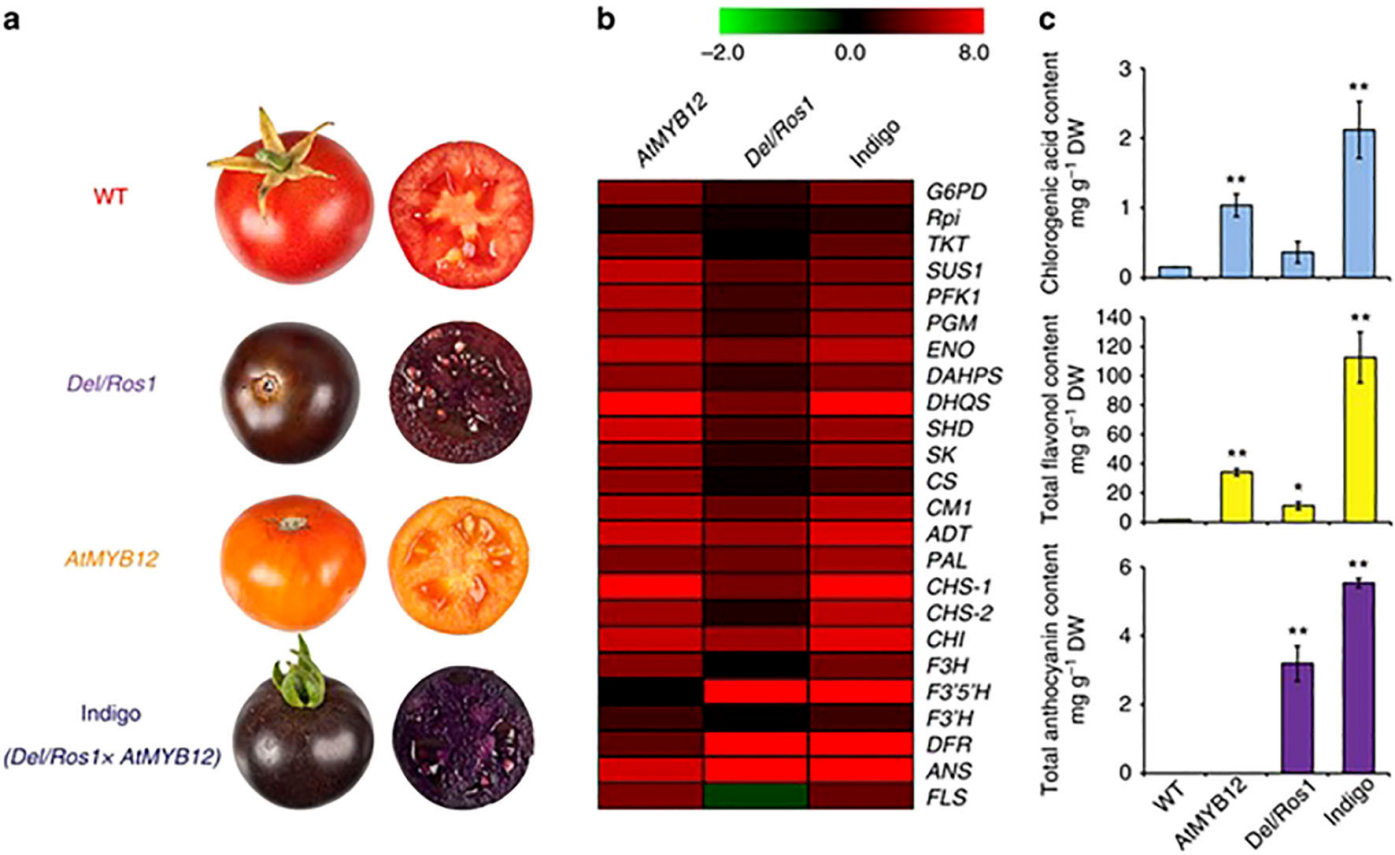
Flavonoid content and genes used in biosynthesis in different tomato phenotypes (Zhang et al., 2015). Where **(a)**: tomato phenotypes; **(b)**: RT-qPCR; **(c)**: content of the phenylpropanoids. * significant value, ** high significant value.

There are a lot of transcription factors (TFs), hormones, and environmental signals that work together to control the transcription of flavonoid biosynthesis. The dynamic MBW (MYB-bHLH-WD40) complexes that are formed by the proteins MYB, bHLH (basic Helix-Loop-Helix), and WD40 regulate the expression of structural genes in the flavonoid pathway, such as flavonol synthase (FLS), chalcone synthase (CHS), and flavonoid 3-hydroxylase (F3H). Recent studies highlight the role of R2R3-MYB TFs (e.g., *SlMYB12*, *SlMYB75*) in activating flavonol and anthocyanin biosynthesis, while ethylene and jasmonic acid signaling further fine-tune flavonoid accumulation (Xu et al., 2015). Light and abiotic stresses (e.g., UV radiation, drought) also influence flavonoid production by altering the expression of key regulators. Epigenetic modifications, such as DNA methylation and histone acetylation, add another layer of control. Advances in CRISPR/Cas9-mediated genome editing and omics technologies (transcriptomics, metabolomics) have deepened our understanding of these regulatory networks. This review synthesizes current knowledge on the transcriptional regulation of flavonoids in tomatoes, emphasizing the potential for metabolic engineering to enhance nutritional value and stress resilience. Unraveling these mechanisms could pave the way for developing improved tomato varieties with enriched flavonoid content for better human health and agricultural sustainability, **Figure 6** shown flavonoid content and genes used in biosynthesis in different tomato phenotypes.

### 5.3 Regulation of vitamin C biosynthesis

The transcriptional regulation of AsA biosynthesis, recycling, and degradation plays a pivotal role in determining its accumulation in tomato fruits. Among the most important enzymes in the AsA metabolic pathway are *GDP-L-galactose phosphorylase (GGP)*, *GDP-D-mannose epimerase* (*GME*), and *L-galactono-1,4-lactone dehydrogenase (GLDH)*. They respond to light, abiotic stress, and hormone cues and are tightly controlled by transcription factors (TFs) such as *SlHY5, SlAREB*, and *SlNAC* (Castro et al., 2023; Asa, 2025). Recent advances in CRISPR/Cas9-mediated genome editing and omics approaches have unveiled novel regulatory networks, providing potential strategies for enhancing vitamin C content in tomatoes.

### 5.4 Epigenetic and post-transcriptional control

There are many layers of control on the manufacture and accumulation of these functional parts in Tomato (*Solanum lycopersicum*), such as epigenetic changes and post-transcriptional regulatory mechanisms. Understanding these regulatory processes is crucial for enhancing the nutritional quality of tomatoes through breeding or biotechnological approaches.

#### 5.4.1 Epigenetic regulation of carotenoids, flavonoids, and Vitamin C

Heritable variations in gene expression that do not result from modifications to the DNA sequence are referred to as epigenetics. DNA methylation and histone alterations are important epigenetic processes, and small RNA-mediated silencing, all of which influence the biosynthesis of health-promoting compounds in tomatoes.

##### 5.4.1.1 DNA methylation and carotenoid biosynthesis

Carotenoids, such as lycopene and β-carotene, are synthesized via the methylerythritol phosphate (MEP) and carotenoid pathways, with key genes like *PSY1* (phytoene synthase 1) and *LCYB* (lycopene β-cyclase) playing pivotal roles. DNA methylation in promoter regions can suppress or activate carotenoid-related genes (e.g., hypomethylation of the *PSY1* promoter is associated with increased lycopene accumulation in ripening tomatoes) (Liu et al., 2015b, 2016, 2015a). Environmental factors (light, temperature) influence methylation patterns, affecting carotenoid levels.

##### 5.4.1.2 Histone modifications and flavonoid production

Flavonoids, including quercetin, kaempferol, and naringenin chalcone, are regulated by the phenylpropanoid pathway, with genes such as *CHS* (chalcone synthase) and *FLS* (flavonol synthase) being critical. Histone acetylation (e.g., H3K9ac) and methylation (H3K4me3) enhance the expression of flavonoid biosynthetic genes (e.g., the MYB12 transcription factor, which activates flavonoid biosynthesis, is epigenetically regulated by histone modifications) (Czemmel et al., 2017). Polycomb-group proteins can repress flavonoid genes via *H3K27me3* marks under certain developmental conditions.

##### 5.4.1.3 Small RNAs and vitamin C regulation

Vitamin C (ascorbate) biosynthesis involves the Smirnoff-Wheeler pathway, with *GGP* (GDP-L-galactose phosphorylase) being a key enzyme. MicroRNAs (miRNAs) and small interfering RNAs (siRNAs) modulate ascorbate levels by degrading mRNA or inhibiting translation (e.g., *miR398* targets *GGP*, reducing ascorbate accumulation under oxidative stress) (Lin et al., 2014). DNA methylation in the *GalUR* (L-galactono-1,4-lactone dehydrogenase) promoter can alter vitamin C content.

#### 5.4.2 Post-transcriptional control mechanisms

**Figure 7:**
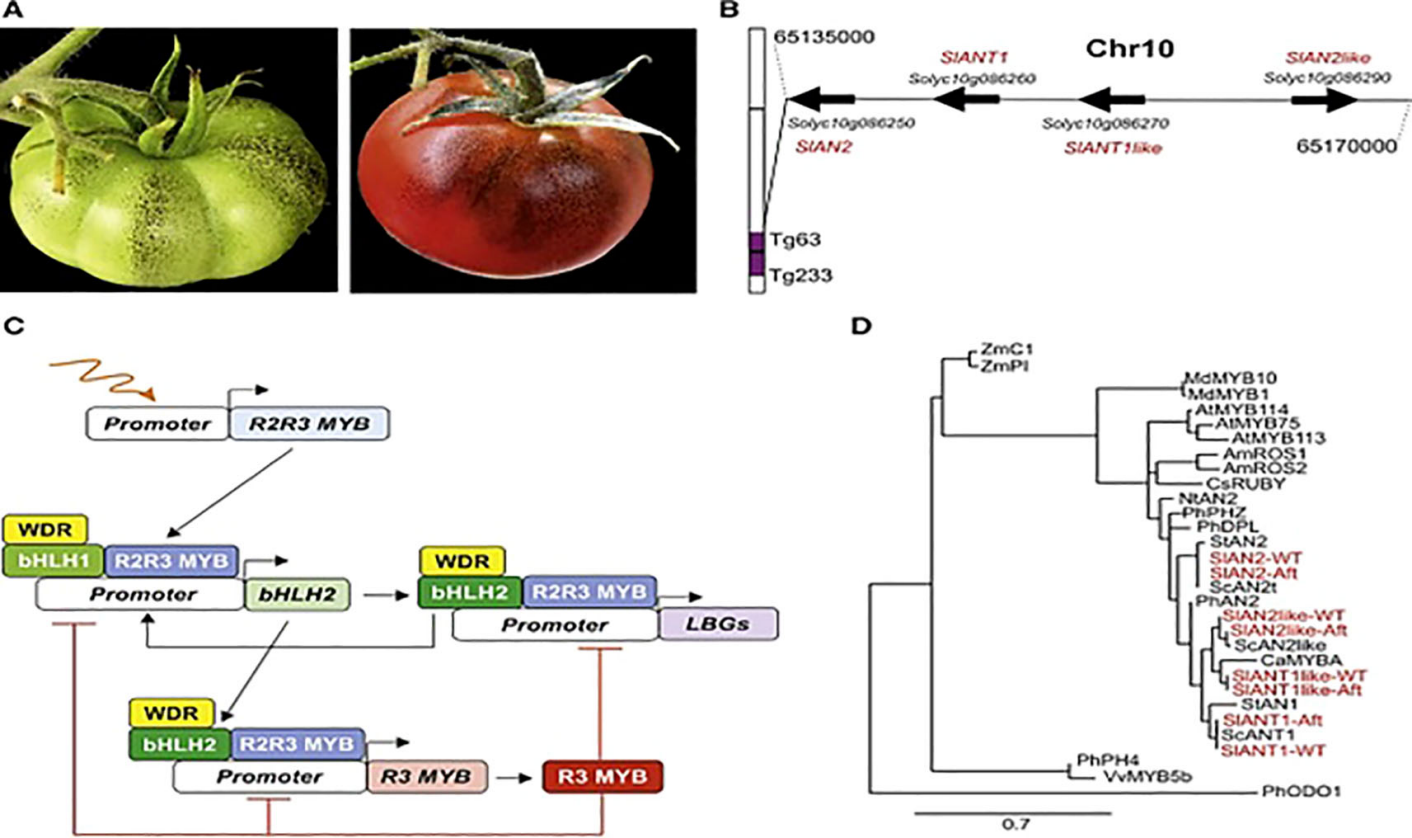
Phytochemicals Synthesis in *Aft* Tomato (Colanero et al., 2020). Where **(A)** *Aft* tomato fruit at mature and ripening stage; **(B)** Chromosome 10 had encoding genes; **(C)** Regulatory mechanism of phytochemicals synthesis; **(D)** Phylogenetic tree among R2R3 MYB proteins and R2R3 MYB factors.

Post-transcriptional regulation fine-tunes metabolite levels through RNA stability, alternative splicing, and translational control as shown in **Figure 7**.

##### 5.4.2.1 Alternative splicing and carotenoid diversification

By generating many mRNA isoforms from a single gene, alternative splicing (AS), a crucial post-transcriptional regulatory step, enhances proteome diversity (Manuel et al., 2023). In plants, AS plays a pivotal role in developmental processes, stress responses, and metabolic regulation (Staiger and Brown, 2013). Carotenoids, essential pigments involved in photosynthesis, photoprotection, and phytohormone synthesis, contribute significantly to fruit quality and nutritional value in tomato (*Solanum lycopersicum*) (Perveen et al., 2015). Recent studies have revealed that AS modulates key genes in the carotenoid biosynthesis pathway, influencing carotenoid composition and accumulation. For instance, differential splicing of genes such as *PSY1* (Phytoene Synthase 1), *LCY-B* (Lycopene Beta-Cyclase), and *CCD* (Carotenoid Cleavage Dioxygenase) generates transcript variants with distinct functional properties, thereby fine-tuning carotenoid profiles (Ampomah-Dwamena et al., 2022; Palaniswamy et al., 2024). Understanding the role of AS in carotenoid metabolism provides novel insights into the molecular mechanisms underlying fruit ripening and offers promising strategies for crop improvement.

##### 5.4.2.2 miRNA-mediated regulation of flavonoids

The flavonoids biosynthesis is tightly regulated at transcriptional and post-transcriptional levels, with emerging evidence highlighting the role of microRNAs (miRNAs) as key modulators. Gene expression is controlled by translational suppression or cleavage of mRNAs, which is targeted by small non-coding RNAs known as miRNAs (Bawazeer, 2022; Ma et al., 2022). Recent studies have identified several miRNAs (e.g., miR156, miR828, miR858) that regulate flavonoid pathways by targeting transcription factors (*MYB*, *bHLH*, *WD40*) and structural genes (*CHS*, *F3H*, *DFR*). For instance, miR858 suppresses *SlMYB7*, a positive regulator of anthocyanin biosynthesis, while miR156 modulates flavonol accumulation by targeting *SPL* genes (Yang et al., 2022; Sun et al., 2017). Environmental stresses, such as UV radiation and nutrient deficiency, alter miRNA expression, further influencing flavonoid profiles. Understanding miRNA-mediated regulation provides novel biotechnological avenues for enhancing tomato nutritional value and stress adaptation through miRNA manipulation or CRISPR-based editing.

##### 5.4.2.3 RNA editing and vitamin C homeostasis

RNA editing is a crucial post-transcriptional mechanism that introduces nucleotide modifications in RNA molecules, enriching transcriptomic and proteomic diversity (Nachtergaele and He, 2017). In plants, this process predominantly involves cytidine-to-uridine (C-to-U) conversions (Bhakta and Tsukahara, 2022), mediated by the pentatricopeptide repeat (PPR) protein family and associated factors. Recent studies suggest that RNA editing plays a vital role in plant development, stress responses, and metabolic regulation. One such metabolic pathway influenced by RNA editing is Vitamin C (ascorbate) homeostasis, a key antioxidant system in plants that mitigates oxidative damage and supports growth. In tomato (*Solanum lycopersicum*), Vitamin C biosynthesis occurs primarily via the D-mannose/L-galactose pathway, with GDP-L-galactose phosphorylase (GGP) being a rate-limiting enzyme (Mellidou et al., 2021). Emerging evidence indicates that RNA editing may fine-tune the expression or functionality of genes involved in ascorbate metabolism, potentially impacting fruit nutritional quality and stress resilience. However, the precise interplay between RNA editing and Vitamin C regulation in tomato remains underexplored. This study investigates the potential crosstalk between RNA editing events and Vitamin C homeostasis in tomato by analyzing RNA-editing patterns in genes related to ascorbate biosynthesis, recycling, and degradation. Utilizing high-throughput RNA sequencing and bioinformatics tools, we identify conserved editing sites in transcripts encoding GGP, ascorbate peroxidase (APX), and monodehydroascorbate reductase (MDHAR). Furthermore, we assess how RNA editing modulates enzyme efficiency and transcript stability under varying ascorbate levels. Our findings reveal that RNA editing dynamically influences key regulatory nodes in Vitamin C metabolism, suggesting a novel layer of post-transcriptional control. These insights could give the way for enhancing tomato fruit nutritional quality through targeted manipulation of RNA editing mechanisms. Understanding this interplay may also have broader implications for improving stress tolerance and antioxidant capacity in crops.

Epigenetic and post-transcriptional mechanisms play a crucial role in controlling carotenoids, flavonoids, and vitamin C in tomatoes. Advances in omics technologies (epigenomics, transcriptomics) and genome editing provide powerful tools to manipulate these pathways for improved nutritional content. Future research should focus on tissue-specific epigenetic marks and stress induced post-transcriptional regulation to develop climate-resilient, nutrient-dense tomato varieties.

## 6 Health benefits of tomato bioactive compounds

**Figure 8:**
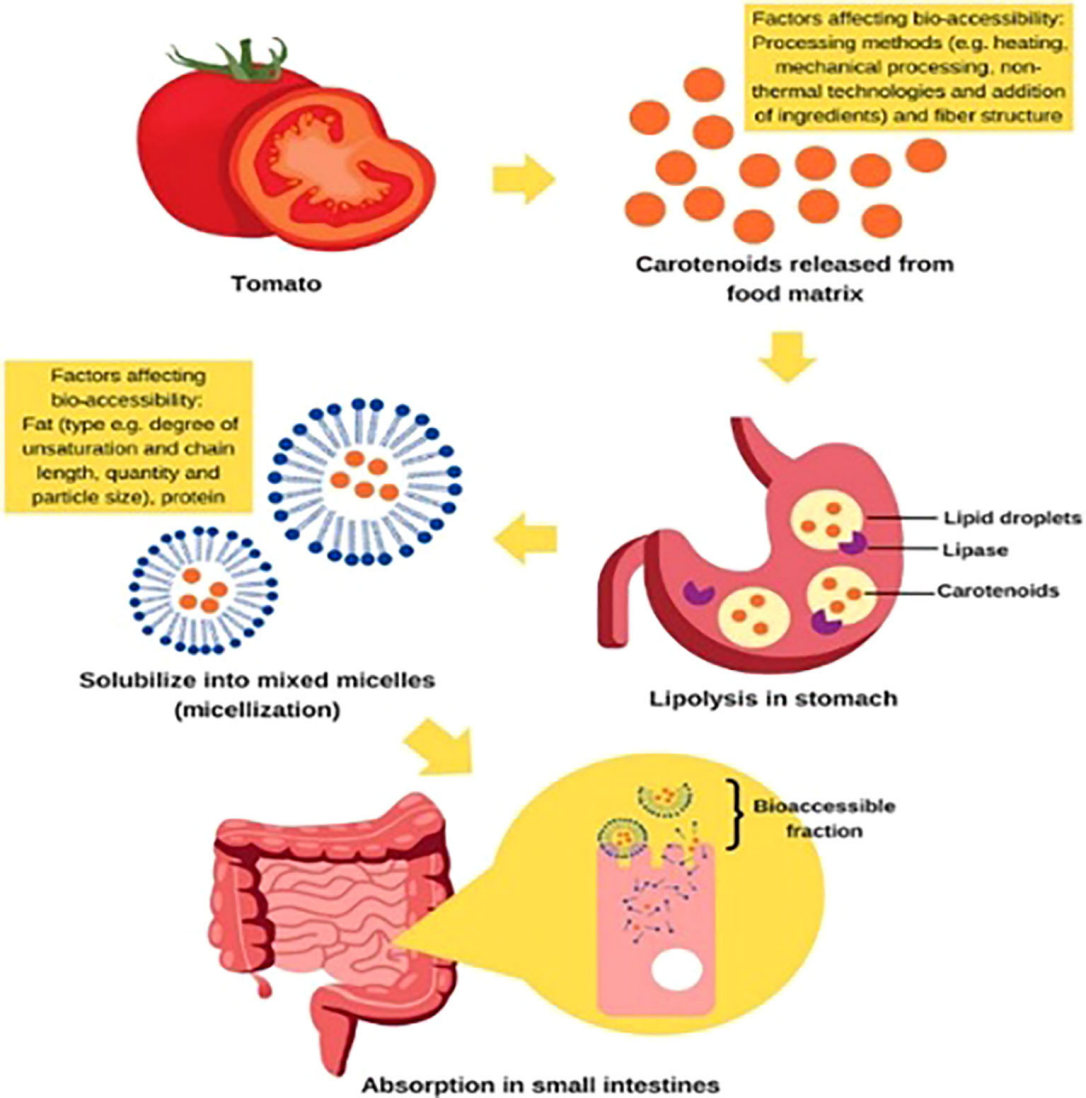
Factors affecting the bio-accessibility and extraction of carotenoids during the human digestion process.

Research on functional phytochemicals in tomatoes holds profound potential for societal impact by directly addressing the escalating burden of chronic diseases and fortifying public health. Tomatoes are a rich source of bioactive compounds like lycopene, flavonoids, and vitamin C, which have demonstrated potent antioxidant and anti-inflammatory properties. By elucidating how these compounds can help mitigate the risk of major conditions such as cardiovascular disease, certain cancers, and neurodegenerative disorders, this research can inform evidence-based dietary guidelines and public health campaigns. Encouraging the consumption of tomato-based products or guiding the breeding of more nutritious varieties could empower individuals with accessible, food-based strategies for prevention. This shift from treatment to proactive, dietary-based wellness has the potential to reduce healthcare costs, improve quality of life, and alleviate the significant economic and social strains that chronic illnesses place on communities worldwide. The antioxidant, anti-inflammatory, anticancer, cardioprotective, and neuroprotective properties of tomatoes’ bioactive compounds which include carotenoids (lycopene, β-carotene), flavonoids, phenolic acids, vitamins C, E, and K, and glycoalkaloids have a substantial positive impact on human health (Chaudhary et al., 2018; Collins et al., 2022; Piccolo et al., 2024). **Figure 8** illustrates some of the variables influencing the bio-accessibility and extraction of carotenoids during the digestive process in humans. Their bioavailability during human digestion, several factors affect the proportion that is liberated from the food matrix and made available for intestinal absorption. This review examines the key determinants affecting carotenoid bio-accessibility and extraction, including food matrix properties (cell wall structure, particle size, and mechanical processing); digestive conditions (gastric pH, enzymatic activity, bile salts, and lipid co-ingestion); and molecular interactions (binding with proteins, dietary fiber, and encapsulation systems).

The food matrix plays a crucial role, as carotenoids in plant chromoplasts are often bound to proteins or fibers, requiring mechanical (e.g., chewing, homogenization) and thermal processing to enhance release (Raikos, 2017). Digestive factors, such as gastric lipase activity and emulsification by bile salts, significantly influence carotenoid solubilization in mixed micelles, which is essential for absorption (McClements, 2018). The presence of dietary lipids (5–10 g per meal) is critical for carotenoid dissolution, while excessive fiber may hinder micelle formation (Verkempinck, 2018). Furthermore, processing techniques (cooking, high-pressure homogenization, and fermentation) can disrupt cell walls, improving carotenoid extractability. Understanding these factors is essential for optimizing dietary strategies and food processing methods to enhance carotenoid bioavailability.

### 6.1 Key bioactive compounds in tomatoes and their health benefits

#### 6.1.1 Lycopene: the potent antioxidant

Lycopene, a red carotenoid pigment, is the most studied bioactive compound in tomatoes, responsible for their vibrant color. It exhibits strong antioxidant properties, neutralizing free radicals and reducing oxidative stress, which is linked to chronic diseases. The health benefits of lycopene are (Przybylska, 2020; Del Giudice et al., 2017; Imran et al., 2020); cardiovascular protection reduces LDL cholesterol oxidation, lowers atherosclerosis risk, improves endothelial function and reduces blood pressure, inhibits platelet aggregation, and reducing thrombosis risk. Anticancer properties reduce prostate cancer risk by modulating cell proliferation and apoptosis. May protect against breast, lung, and stomach cancers due to its anti-inflammatory effects. Skin health protects against UV-induced skin damage by scavenging free radicals. May delay skin aging by improving collagen synthesis. Bone health reduces oxidative stress in bones, potentially lowering osteoporosis risk.

#### 6.1.2 β-carotene: provitamin A activity

Tomatoes contain β-carotene, a precursor to vitamin A, essential for vision, immune function, and skin health. The health benefits of β-Carotene (Tufail et al., 2024; Akram et al., 2021); enhances immune function supports mucosal immunity and reduces infection risk. Eye Health prevents age-related macular degeneration (AMD) and night blindness. Antioxidant effects work synergistically with lycopene to combat oxidative stress.

#### 6.1.3 Flavonoids (quercetin, kaempferol, naringenin)

Tomatoes are rich in flavonoids, which have anti-inflammatory and anticancer properties (Cuevas-Cianca et al., 2023; Ba et al., 2023). The health benefits of flavonoids are anti-inflammatory effects inhibiting pro-inflammatory cytokines (e.g., TNF-α, IL-6). Cardioprotective improves vascular function and reduces arterial stiffness. Antidiabetic effects enhance insulin sensitivity and reduce blood glucose levels.

#### 6.1.4 Vitamin C (ascorbic acid)

Tomatoes provide a significant amount of vitamin C, an essential water-soluble antioxidant (Muzolf-Panek et al., 2017). The health benefits of Vitamin C are boosts immunity stimulates white blood cell production. Collagen synthesis is essential for wound healing and skin elasticity. Enhances iron absorption reduces anemia risk by improving non-heme iron uptake.

#### 6.1.5 Phenolic acids (chlorogenic acid, coumaric acid)

These compounds contribute to tomato’s antioxidant capacity and disease prevention. The health benefits of phenolic acids are (El-Nagar et al., 2020; Zárate-Martínez et al., 2021); neuroprotective Effects may reduce Alzheimer’s and Parkinson’s disease risk by preventing oxidative neuronal damage. Antidiabetic Properties inhibit carbohydrate-digesting enzymes, lowering postprandial glucose spikes.

#### 6.1.6 Glycoalkaloids (tomatine)

Though toxic in high doses, tomatine in moderate amounts has antimicrobial and cholesterol-lowering effects (Abdul Latif et al., 2018). The health benefits of glycoalkaloids are antimicrobial activity effective against bacteria and fungi. Cholesterol reduction binds to cholesterol in the gut, reducing absorption.

**Figure 9:**
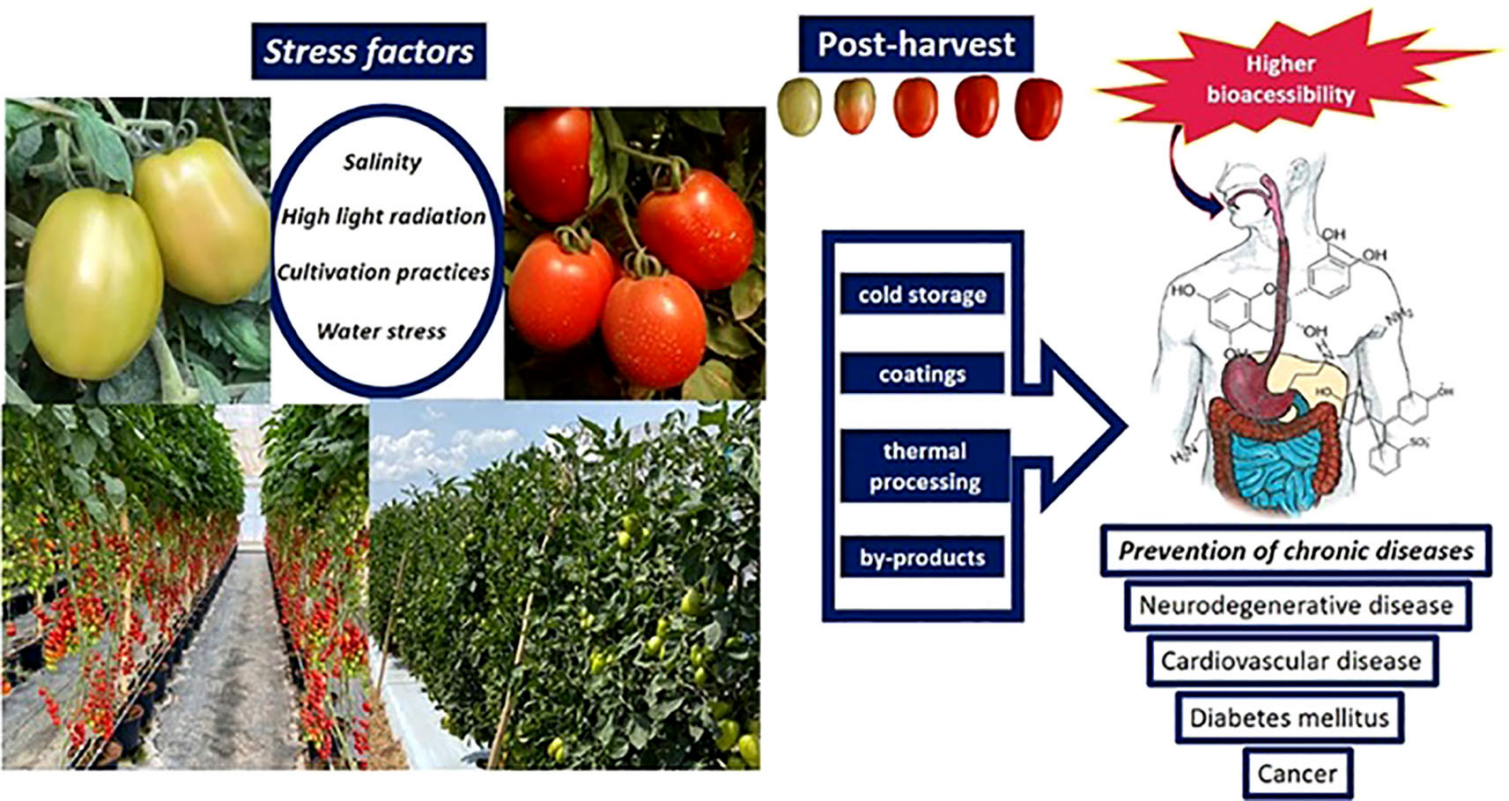
Impact of biotic and abiotic variables on tomato bioactive components’ bio-accessibility and bioavailability.

Tomatoes are a powerhouse of bioactive compounds with multifaceted health benefits, ranging from cardiovascular protection to cancer prevention. Regular consumption, especially in processed forms with healthy fats, maximizes their therapeutic potential. Further research continues to uncover novel mechanisms by which these compounds promote health, reinforcing tomatoes as a functional food in disease prevention and longevity, **Figure 9** shows the impact of biotic and abiotic variables on tomato bioactive components’ bio-accessibility and bioavailability.

## 7 Conclusion and future perspectives

Carotenoids (lycopene, β-carotene), flavonoids, phenolic acids, and glycoalkaloids are among the essential nutritional components of tomatoes (*Solanum lycopersicum*), which have a major positive impact on human health. These bioactive compounds exhibit anti-inflammatory, antioxidants, anticancer, and cardioprotective properties, making them crucial in preventing chronic diseases such as cardiovascular disorders, and diabetes. The biosynthesis of these phytochemicals is tightly regulated by genetic and environmental factors, with key genes (e.g., *PSY*, *LCY*, *MYB*, *HY5*) playing pivotal roles in their metabolic pathways. Recent developments in transcriptomics, metabolomics, and genomes have expanded our knowledge of the molecular processes that underlie the synthesis and control of phytochemicals. CRISPR-Cas9 and other gene-editing technologies offer promising avenues for enhancing the nutritional content of tomatoes by modulating biosynthetic pathways. In summary, the tomato transcends its role as a dietary staple to emerge as a powerful nexus of agricultural science, molecular biology, and human health. The profound health benefits conferred by its diverse phytochemical portfolio are undeniable, yet we stand only at the frontier of fully harnessing this potential. By systematically decoding and optimizing the tomato’s genetic blueprint and its interaction with the environment, we can purposefully engineer this vital crop into a more potent, natural preventative healthcare solution. Ultimately, the goal is to transform this everyday fruit into a cornerstone of future functional foods, capable of delivering targeted, scientifically validated health benefits to populations worldwide, thereby redefining the connection between diet and disease prevention. The compelling evidence for the diverse health-promoting properties of functional phytochemicals in tomatoes, from the cardioprotective effects of lycopene to the antioxidative power of various flavonoids and carotenoids, must not remain confined to academic journals. To truly unlock the potential of the tomato as a functional food, a concerted and collaborative effort is urgently needed. We therefore issue a strong call to action for researchers, agricultural scientists, food technologists, and industry stakeholders to bridge the gap between foundational science and tangible application. By investing in and collaborating on the identified research areas; such as optimizing cultivation practices, enhancing bioavailability, and developing novel, evidence-based food products, we can collectively translate this promise into a new generation of health-focused foods. Let us harness this synergistic potential to not only advance scientific understanding but also to deliver meaningful health benefits to consumers worldwide.
