# Correction: Functional phytochemicals in tomatoes: biosynthesis, gene regulation, and human health implications

**DOI:** 10.3389/fpls.2026.1798850

**Published:** 2026-02-24

**Authors:** Essam ElShamey, Yawen Zeng, Yumei Ding, Jiazhen Yang

**Affiliations:** 1 Biotechnology and Germplasm Resources Institute, Yunnan Academy of Agricultural Sciences, Kunming, China; 2 Rice Research Department, Field Crops Research Institute, Agricultural Research Center, Giza, Egypt; 3 College of Food Science and Technology, Yunnan Agricultural University, Kunming, China

**Keywords:** tomato, functional components, biosynthesis pathways, gene regulation, health benefits, carotenoids, flavonoids

There was a mistake in the caption of **Figure 4** as published. The citation “(Mellidou et al., 2021)” was omitted. The caption of **Figure 4** has been updated to “**Figure 4**. Schematic biosynthesis pathways for vitamin C in tomato fruits (Mellidou et al., 2021)”.

The reference “Mellidou, I., Koukounaras, A., Kostas, S., Patelou, E., and Kanellis, A. K. (2021). Regulation of vitamin C accumulation for improved tomato fruit quality and alleviation of abiotic stress. Genes. 12, 694. doi: 10.3390/genes12050694” which was omitted from the published version of the article, has now been added to the reference list.

The original version of this article has been updated.

